# Urine is a novel source of autologous mesenchymal stem cells for patients with epidermolysis bullosa

**DOI:** 10.1186/s13104-015-1686-7

**Published:** 2015-12-10

**Authors:** Markus Schosserer, Rita Reynoso, Verena Wally, Bogdan Jug, Viktoria Kantner, Sylvia Weilner, Ivana Buric, Johannes Grillari, Johann W. Bauer, Regina Grillari-Voglauer

**Affiliations:** Department of Biotechnology, University of Natural Resources and Life Sciences, Vienna, Muthgasse 18, 1190 Vienna, Austria; Division of Experimental Dermatology, EB House Austria, Department of Dermatology, Paracelsus Medical University Salzburg, Muellner Hauptstrasse 48, 5020 Salzburg, Austria; Evercyte GmbH, Muthgasse 18, 1190 Vienna, Austria; Christian Doppler-Laboratory on Biotechnology of Skin Aging, 1190 Vienna, Austria; Austrian Cluster for Tissue Regeneration, Vienna, Austria

**Keywords:** Urine-derived stem cells (USCs), Epidermolysis bullosa (EB), Stem cell differentiation, Immune-modulatory properties

## Abstract

**Background:**

Regenerative medicine is strictly dependent on stem cells as a source for a high diversity of somatic cells. However, the isolation of such from individuals suffering from severe genetic skin blistering diseases like epidermolysis bullosa (EB) is often associated with further organ damage.

**Methods:**

Stem cells were isolated from 112 urine samples from 21 different healthy donors, as well as from 33 urine samples from 25 donors with EB. The cultivation of these cells was optimized by testing different media formulations and pre-coating of culture vessels with collagen. The identity of cells was confirmed by testing marker expression, differentiation potential and immune-modulatory properties.

**Results:**

We provide here an optimized protocol for the reproducible isolation of mesenchymal stem cells from urine, even from small volumes as obtained from patients with EB. Furthermore, we offer a basic characterization of those urine-derived stem cells (USCs) from healthy donors, as well as from patients with EB, and demonstrate their potential to differentiate into chondrocytes, osteoblasts and adipocytes, as well as their immune-modulatory properties.

**Conclusions:**

Thus, USCs provide a novel and non-invasive source of stem cells, which might be applied for gene-therapeutic approaches to improve medical conditions of patients with EB.

**Electronic supplementary material:**

The online version of this article (doi:10.1186/s13104-015-1686-7) contains supplementary material, which is available to authorized users.

## Background

Epidermolysis bullosa (EB) is a devastating disease of the skin and mucous membranes, caused by mutations in either one of 17 genes encoding structural proteins required for skin integrity. Patients suffer from extensive blistering upon minor mechanical impact due to the reduced connectivity of the skin layers. In severe subtypes of EB, the continuous wounding of the skin causes a constant need for repair, which leads to the depletion of epidermal stem cells and thus to multiple chronic wounds [1].

Currently, ex vivo gene therapy is the most promising therapeutic approach concerning safety and technical realization. Mavilio et al. [2] described the transplantation of in vitro engineered skin grafts (500 cm^2^ in total) onto the backside of the legs of a patient with EB. Epidermal stem cells were isolated from patient skin biopsies, retrovirally transduced with a wildtype copy of the defective laminin β3 (LAMB3) cDNA in a MFG vector, and expanded in vitro. Clinically, the patient showed no more blistering in the area of the transplanted skin until 6.5 years after the transplantation, proving the long-term stability and safety of this approach [3]. However, the recovery of pertinent epidermal stem cells, which usually requires multiple skin biopsies, is an extremely displeasing and painful demand especially for patients with EB. Thus, other sources for the non-invasive recovery of stem cells, such as urine, are required.

Zhang and co-workers recently described the isolation of mesenchymal-like stem cells from urine. These multi-potent cells are termed urine-derived stem cells (USCs) and were shown to have the potential to differentiate towards osteocytes, chondrocytes, adipocytes, myocytes and endothelial cells [4–11]. Mesenchymal lineages, such as fibroblasts, could be used for the treatment of patients suffering from dystrophic EB. Furthermore, recent reports suggest that mesenchymal stem cells can also be trans-differentiated into epidermal cells [12, 13] which might be beneficial for the treatment of the other subtypes. Thus, the isolation of USCs from urine might provide a non-invasive route for the painless recovery of stem cells from patients with EB.

We show here that by using an optimized protocol USCs can be reproducibly isolated from small volumes of urine from healthy donors, as well as from patients with EB. Recovered USCs were positive for various mesenchymal marker genes and could be differentiated into osteocytes, chondrocytes and adipocytes. Furthermore USCs showed similar immunomodulatory properties as amnion-derived mesenchymal stem cells.

## Methods

### Ethics

The isolation USCs from urine samples from healthy donors within this study was reviewed and approved by the ethic commission of the Medical University of Vienna (Ethik-Kommission der Medizinischen Universität Wien, Borschkegasse 8b/6, A-1190 Vienna, Austria).

For the isolation of USCs from patients with EB, the Salzburg ethics committee (Ethikkommission für das Bundesland Salzburg, Sebastian-Stief-Gasse 2, A-5020 Salzburg, Austria) waived the formal review of this study by the ethics committee, since no clinical trials were conducted. This procedure is in accordance with the Krankenanstalten-und Kuranstaltengesetz, §8c (Austrian Federal Hospital Act, section 8c), and with the Salzburger Krankenanstaltengesetz 2000, §30 (Salzburg Hospital Act 2000, section 30).

All the donors gave informed consent before providing urine samples. Thus, the study was performed in accordance with the Declaration of Helsinki.

### Isolation of USCs from urine

Mid- and last-stream urine from healthy donors was collected into sterile recipes, transferred to 50 ml tubes and centrifuged for 5 min at 500×*g*. Thereafter, the supernatant was discarded and cells were washed twice with PBS. The remaining pellet was re-suspended in culture medium and seeded into one well of a 24-well plate for male donors or a 6-well plate for female donors. Culture plates (Nunc) were either used without coating or coated with Collagen (Sigma Aldrich). Four different formulations of culture medium were used:

1. Primary urine cells culture medium [pUSCs, according to the method described by Zhang et al. [4], 50 % Keratinocyte-SFM (Gibco), 33.75 % DMEM (PAA), 11.25 % Hams F12, 5 % Fetal calf serum (FCS, PAA), 5 ng/ml Epidermal growth factor (Sigma Aldrich), 50 ng/ml Bovine pituitary extract (Sigma Aldrich), 30 ng/ml Cholera toxin (Sigma Aldrich), 0.4 μg/ml Hydrocortisone (Sigma Aldrich), 5 ng/ml Insulin (Sigma Aldrich), 1.8 × 10^−4^ M Adenine (Sigma Aldrich), 5 μg/ml Holo-Transferrin (VWR), 2 nM Triiodo-L-thyronine (Life Technologies)].
2. MesenCult^®^ MSC Basal Medium supplemented with MesenCult™ Mesenchymal Stem Cell Stimulatory Supplement (Stem Cell).
3. MesenPRO RS™ supplemented with MesenPRO RS™ Growth Supplement (Gibco).
4. EGM™-2 Bulletkit (Lonza).

All formulations were additionally supplemented with 100 U/ml penicillin and 100 µg/ml streptomycin (Sigma).

Urine pH was measured and samples from healthy donors were also evaluated for the presence of glucose, leucocytes, nitrites, proteins and blood using Combur Test HC (Cobas) [14].

### Immunophenotypic characterization of USCs by fluorescence microscopy

After three or four passages, USCs and adipose derived stem cells (ASCs) as control, were seeded into 8 well chamber slides (µ-Slide, IBIDI-Treat surface, IBIDI) without coating. Next day, cells were rinsed with PBS and fixed with 3.6 % formaldehyde in PBS for 10 min at room temperature. After two consecutive washes with PBS, the cells were stained for 1 h at room temperature with antibodies against CD14 (from mouse, ab7800, Abcam), CD34 (from mouse, FITC-labeled, 555821, BD Pharmingen), CD44 (from mouse, SAB4700184, Sigma Aldrich), CD73 (from mouse, FITC-labeled, 561254, BD Pharmingen), CD90 (from mouse, PE-labeled, 555596, BD Pharmingen), CD105 (from mouse, FITC-labeled, 561443, BD Pharmingen), CD117 (from mouse, FITC-labeled, 561443, BD Pharmingen), CD133 (from rabbit, ab19898, Abcam), Vimentin (from mouse, FITC-labeled, 562338, BD Pharmingen), and Collagen I (from rabbit, ab292, Abcam). For antibodies without dye-conjugate, samples were washed three times and incubated with Dyelight488 or Dyelight549-conjugated secondary antibodies against mouse or rabbit IgG (Jackson Immunoresearch) for 1 h. Finally, the wells were washed twice with PBS, stained with Hoechst 33258 for 5 min, washed once with PBS and analyzed by fluorescence microscopy on a Leica DMI-6000 microscope equipped with filter sets for DAPI, GFP and RFP.

### Immunophenotypic characterization of USCs by flow cytometry

After three or four passages, USCs, as well as adipose derived stem cells (ASCs) and mesenchymal stem cells from umbilical cord [MSCs (UC)] as control, were harvested and fixed with 70 % Ethanol overnight at 4 °C. After two consecutive washes with PBS, the cells were stained for 1 h at room temperature with antibodies against CD73 (from mouse, FITC-labeled, 561254, BD Pharmingen) and CD90 (from mouse, PE-labeled, 555596, BD Pharmingen). Cells were washed thrice in PBS and analyzed on a Gallios Flow Cytometer (Backman Coulter).

### Cell differentiation

For osteogenic differentiation, 3000 cells were seeded into one well of a 24-well plate without coating containing 1 ml DMEM low glucose (PAA) supplemented with 10 % FCS, 10 nM dexamethasone (Sigma), 150 μM ascorbate 2-phosphate (Sigma), 10 nM vitamin D3 (Sigma) and 10 mM l-glycerophosphate (Sigma). The plates (Nunc) were kept in a humidified incubator (37 °C and 5 % CO_2_) and the culture medium was changed twice a week for 28 days.

For healthy donors, the medium was aspirated after this time and the cells were washed three times with PBS. Then, cells were fixed in ice-cold 70 % ethanol for 1 h at −20 °C and washed three times with distillated water. The cells were incubated with 40 mM Alizarin Red solution (Sigma) at room temperature for 10 min and washed until all remaining traces of dye were removed. Representative pictures were acquired on a Leica DMI microscope equipped with a color-camera.

Cells differentiated from patients with EB were equilibrated in an alkaline-phosphatase (AP)-buffer (0.1 M Tris–HCl pH 9.5, 0.1 M NaCl, 0.05 M MgCl_2_) for 10 min. Then, cells were incubated in 2 % NBT/BCIP (Sigma) in AP-buffer for 75 min and representative microscope pictures were acquired.

For chondrogenic differentiation, 3000 cells were seeded in 24-well plates (Nunc) without coating containing 2 ml DMEM/Ham’s F12 supplemented with 10 % FCS, 6 μg/ml insulin (Sigma), 0.2 mM ascorbic acid-2P (Fluka) and 10 ng/ml TGF-β1 (R&D). The plates (Nunc) were kept in a humidified incubator (37 °C and 5 % CO_2_) and the culture medium was changed twice a week for 28 days.

After this period, the medium was aspirated and the cells were washed three times with PBS. Then, the cells were incubated with 3.6 % formaldehyde in PBS for 1 h, washed three times with distillated water and incubated with 1 % Alcian blue 8GX staining solution (Sigma) at room temperature for 30 min. The cells were washed until all traces of remaining dye were removed and representative microscope pictures were acquired.

For adipogenic differentiation 14,000 USCs or ASCs as positive control were seeded into single wells of 6-well plates in growth medium (DMEM) for 2 days prior to adipogenic induction. Adipogenesis was induced by replacing the growth medium with fresh DMEM supplemented with 549 µM 3-isobutyl-1-methylxanthine (Sigma), 1 µM Dexamethasone (Sigma), 549 µM hydrocortisone (Sigma) and 66 µM Indomethacin (Sigma). Medium was exchanged twice a week until day 24, followed by Oil Red O staining. Differentiation medium was removed and cells were washed briefly with PBS twice and fixed by incubation in 3.6 % formaldehyde in PBS for 1 h at room temperature. Cells were then washed twice for 5 min each with PBS and incubated with 70 % ethanol for 2 min, followed by 10 min incubation in Oil red O working solution (1.8 mg/ml, Sigma). Cells were washed with PBS until all visible traces of remaining dye were removed and representative images were acquired.

Cell differentiations were confirmed by analyzing relative expression levels of different marker genes. Primer sequences are depicted below. Therefore, cells were harvested by trypsinization and total RNA was isolated using QIAzol Lysis Reagent (Qiagen), according to the manufacture’s protocol. RNA pellets were dried and re-suspended in 50 μl RNase-free water. RNA was quantified using a NanoDrop Spectrophotometer (Thermo Scientific) and 100 ng of total RNA were reverse transcribed using Dynamo cDNA synthesis kit (Finnzymes). For quantification of osteocalcin mRNA from healthy donors, qPCR was performed on a Rotorgene Q (Qiagen) using SensiMix Plus SYBR-Green Mix. Expression values were normalized to GAPDH and are indicated as fold changes relative to control.

The relative abundance of Collagen X, Aggrecan and GAPDH mRNAs were evaluated by semi-quantitive real-time PCR (SQRT-PCR). After reverse transcription as above, PCR products were generated using GoTAQ Polymerase (Promega) and separated on a 2 % agarose gel in 1× TBE buffer. PCR conditions were adjusted so that products were not in saturation.

**Table:** 

Target	Primer sense	Primer antisense
GAPDH^a,b^	GCCAACGTGTCAGTGGTGGA	CACCACCCTGTTGCTGTAGCC
Osteocalcin^a^	ATCAAAGAGGAGGGGAACCTA	AGGAAGTAGGGTGCCATAACA
Osteocalcin^b^	GTGCAGAGTCCAGCAAAGGT	TCAGCCAACTCGTCACAGTC
Collagen X^a^	CCCTTTTTGCTGCTAGTATCC	CTGTTGTCCAGGTTTTCCTGGCA
Aggrecan^b^	ACAGCTGGGGACATTAGTGG	GTGGAATGCAGAGGTGGTTT

^a^Primer used for USCs from healthy donors

^b^Primer used for USCs from patients with EB

### Evaluation of immunomodulatory properties

The isolation of peripheral blood mononuclear cells (PBMCs) from whole blood and subsequent lymphocyte separation by plate adherence were performed as described previously [15]. Two different concentrations of USCs (4 × 10^5^ or 2 × 10^5^) were seeded and allowed to attach in individual wells of 6 well-plates (Nunc). Then the medium was replaced with 4 × 10^5^ lymphocytes in RPMI medium supplemented with 10 % FCS and 4 mM glutamine. For the activation of lymphocytes 2.4 μg/ml phytohemagglutinin (PHA, Sigma) were used. After 48 h, proliferation of lymphocytes was determined by bromodeoxyuridine (BrdU) incorporation as described previously [16]. Briefly, 10 μM BrdU were added to the culture medium and lymphocytes were harvested after 24 h. Fixation and permeabilization were followed by staining with anti-BrdU primary antibody (BD Biosciences) and goat-anti-mouse DyLight 488 conjugate secondary antibody (Pierce). Finally, BrdU incorporation was analyzed by flow cytometry (FACSDiva, BD Biosciences).

### Statistical analysis

All the experiments were performed in biological triplicates at least. Correlations were studied using the Pearson test. Association between urine volume and the presence of colonies was determined by Chi Square test. The differences in gene expression, as well as in the number of colonies depending on collagen coating were analyzed by Student’s *t* test. The statistical significance of cell culture characteristics depending on culture medium and lymphocytes proliferation were assessed by one-way ANOVA followed by Bonferroni multiple-comparison post hoc test. All statistical tests were performed in SPSS (IBM). *p* values ≤0.05 were considered significant.

## Results

### Characteristics of donors and urine

In order to establish a robust isolation protocol of USCs in our lab, we obtained in total 112 urine samples from 21 different healthy donors. Only one sample was positive for nitrites, being all of them negative for the presence of glucose, leucocytes, proteins and blood. The volume of urine varied from 50 to 495 ml and the pH from 5.3 to 7.6, however these parameters did not correlate with the presence or quantity of colonies of USCs (*p* = 0.484 and *p* = 0.58, respectively, Pearson Correlation) (Additional file 1: Table S1). These results indicate that neither pH, nor the volume of urine influence the success rate of USCs isolation. Even from small volumes of urine, as usually provided by patients with EB, USCs could be isolated.

### Establishment of a reproducible USCs isolation protocol from healthy donors

**Fig. 1:**
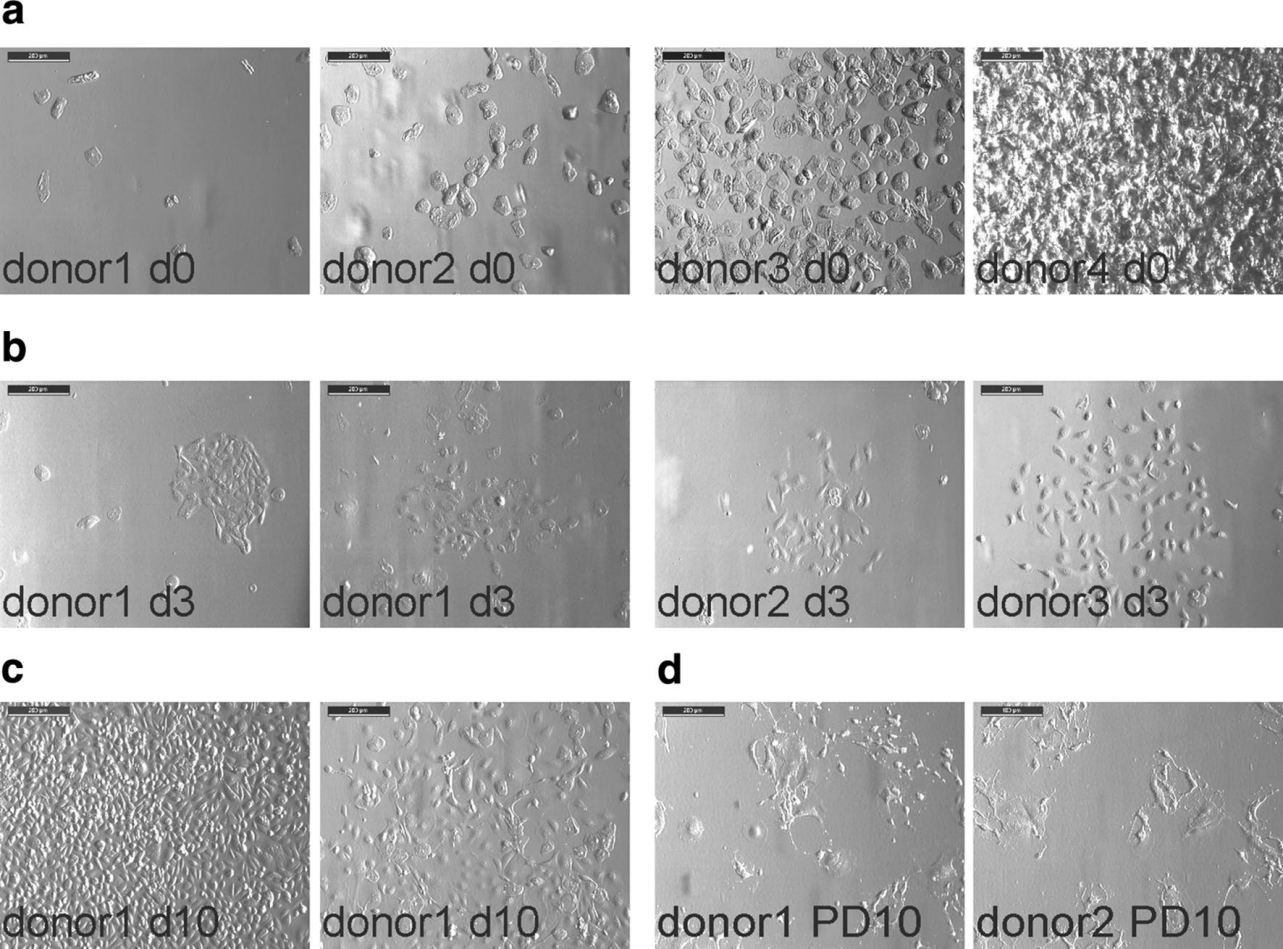
Morphology of USCs. **a** Immediately after seeding (d0), urine samples show different contents of squamous cells, ranging from only few cells to complete coverage of the surface (from *left* to *right panel*). Representative pictures of four different donors are shown. **b** Approximately 3 days after seeding, the formation of colonies could be observed. The variability in size and morphology between donors, as well as between two samples from the same donor are shown. **c** Approximately 10 days (d10) after seeding, the cells reached confluence. Even within two different samples from the same donor, differences in cell morphology could be observed at his stage. **d** After ten PDs on average USCs stopped proliferation and showed the typical senescent morphology. Samples from two different donors are depicted

In order to isolate USCs, urine was centrifuged and cells were seeded in pUSCs medium either in 24-well plates for male donors or 6-well plates for female donors. Urine samples showed a variable content of squamous cells, being in general more abundant in female samples (Fig. 1a). Cell colonies appeared most frequently between 3 and 8 days after isolation. Eventually, samples from specific donors showed the first colony after 2–3 weeks.

In the following we define successful isolation of USCs, when at least one colony was obtained. As shown in Additional file 1: Table S1, the success rate of isolations from male donors was higher than from female donors (70 vs. 42 %). However, we observed that by isolating multiple samples from the same donor within 2 weeks (2–3 samples per day, 3 times per week), at least two successful isolations from each donor could be obtained independent of the donor’s sex.

### Morphology of freshly isolated USCs-enriched cells from urine

Urine derived cells exhibited morphologies ranging from more epithelial to more elongated-fibroblastoid, independent of the donor. This indicates that a mixed population of different cell types was present immediately after isolation. Representative pictures of primary colonies are shown in Fig. 1b. However, after three passages the cell morphology became homogeneously elongated-fibroblastoid in most of the isolations. This cell population homogeneous in morphology is henceforth referred to as USCs and was used for all upcoming experiments. It is unclear, however, if the epithelial cells are lost over passaging due to the media enriching for fibroblastoid cells. The isolated cells reached confluence at around 12 days (Fig. 1c). In general the replicative life span ranged from 4.5 to 12 population doublings following the first passage, after which cells stopped growing and displayed typical senescent morphology (Fig. 1d).

### Optimization of culture conditions

**Fig. 2:**
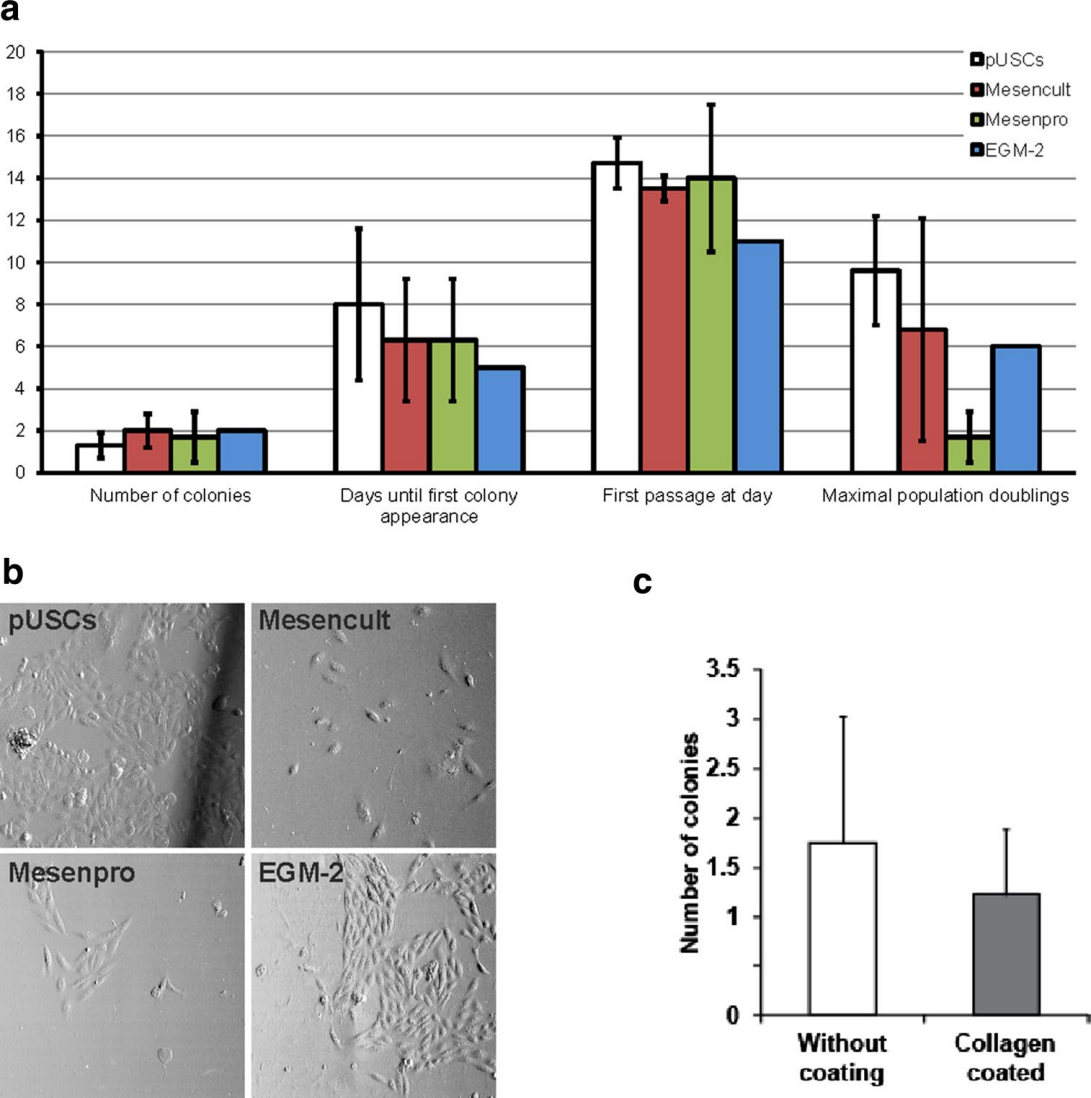
Media optimization for the isolation and cultivation of USCs. **a** Comparison of the effect of different media formulations on the isolation and growth characteristics of USCs. The values represent the mean of at least biological triplicates, except EGM-2, since only one sample showed proliferation in EGM-2. *Error bars* represent the standard deviation. **b** Representative images of cells on day 6–7 after isolation of one donor. **c** Collagen-coating reduces significantly the efficiency of USCs-isolation (*p* = 0.026). *Error bars* indicate the standard deviation of biological triplicates

In order to optimize the culture conditions for USCs, we tested different media formulations. Consequently, urine samples were obtained from four donors and the cells were cultivated either in MesenCult, MesenPRO, EGM-2 or pUSCs medium. We did not see major differences in cell morphology depending on the culture medium used, although cells grown in EGM-2 tended to display a more epithelial phenotype than with the other formulations (Fig. 2a). Cells from only one out of five samples showed cell proliferation in EGM-2 and also in this case cells stopped growing after passage 3. Interestingly, when either MesenCult or MesenPRO medium was used some colonies appeared already as soon as 3 days after isolation. Nevertheless, we did not find significant differences between the four media formulations regarding the necessary timeframe for colony formation. The number of colonies and the time needed to passage the cells for the first time did not differ significantly either (Additional file 1: Table S2). Cells cultivated in MesenPRO or MesenCult stopped cell division after passage 1 in some cases, while in pUSCs medium they grew for longer periods of time and reached significantly more population doublings (PDs) compared to MesenCult (*p* = 0.013) or MesenPRO (*p* = 0.006) (Additional file 1: Table S2).

In addition, we tested whether the pre-coating of culture dishes with collagen improved the efficiency of isolation and culture of USCs. As expected, the number of colonies was significantly higher when the plates were not coated (*p* = 0.026) (Fig. 2b), since adherence to plastic surfaces is typical for mesenchymal stem cells [14].

In summary these results indicate that USCs can be cultivated in different media formulations, but pUSCs medium without collagen coating of culture dishes is most efficient for expanding the cells.

### USCs express typical markers for mesenchymal stem cells (MSCs)

**Fig. 3:**
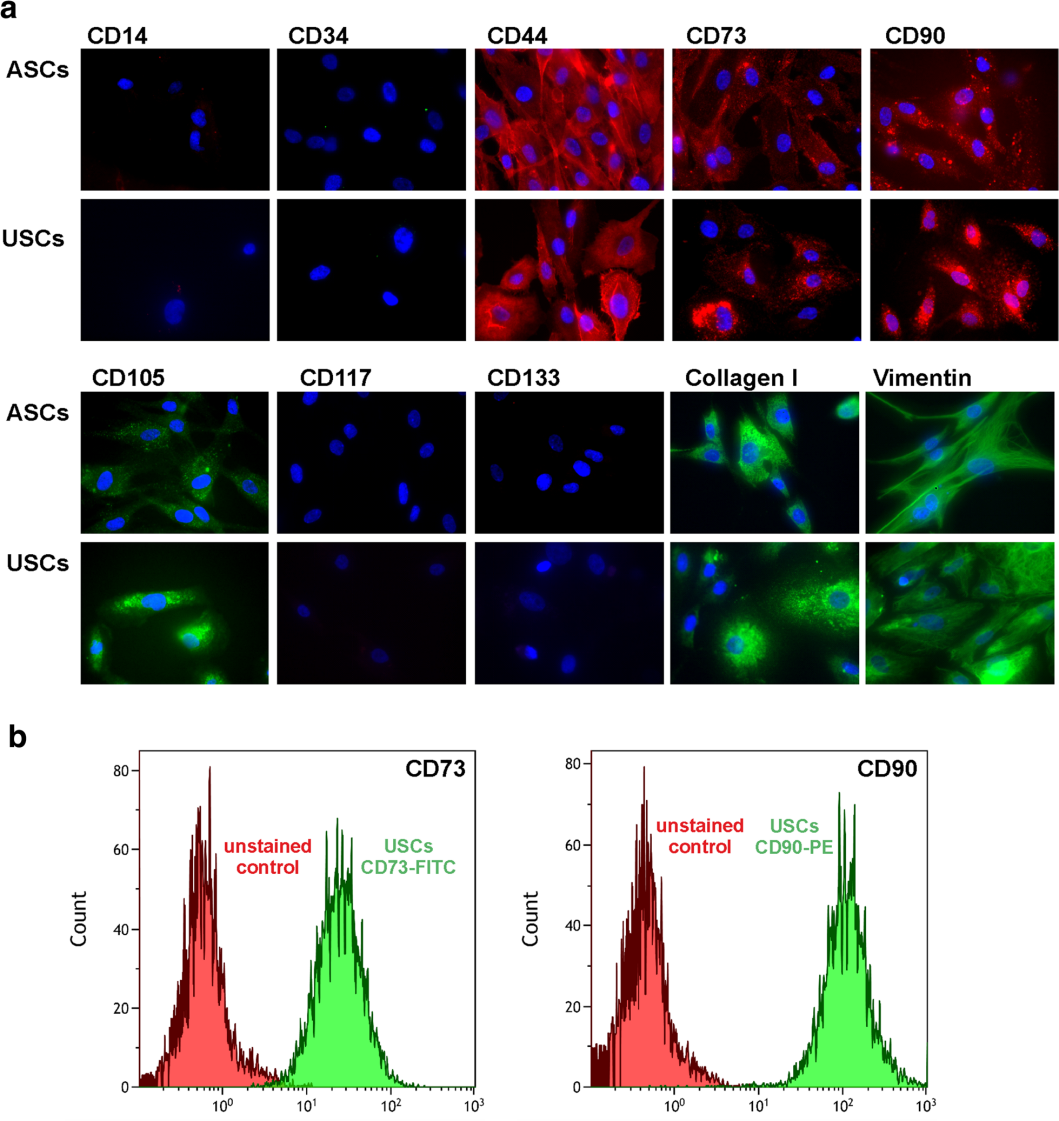
USCs surface marker expression patterns resemble those of ASCs. **a** Representative immunofluorescence images of USCs and ASCs as control are shown. USCs, as well as ASCs, stained positive for the typical MSC markers CD44, CD73, CD90 and CD105, and negative for the hematopoietic markers CD14, CD34, CD117 and CD133. Additionally, the mesenchymal markers Vimentin and Collagen I stained positive both in USCs and ASCs. **b** Representative flow cytometry histograms of USCs stained for CD73 and CD90 reveal a single homogenous population of cells expressing both mesenchymal markers. Two replicates from other donors showed similar results

In order to test if the urine-derived cells are indeed enriched for USCs, the expression of MSC surface markers including CD73, CD90 and CD105 [17], as well as a lack of hematopoietic surface markers was analyzed by indirect immunofluorescence. Adipose derived mesenchymal stem cells (ASCs) served as positive control. Indeed, the surface marker profile of USCs resembled closely that of ASCs, as both were positive for the mesenchymal stem cells markers CD44, CD73, CD90, CD105, Vimentin and Collagen I, and negative for the hematopoietic markers CD14, CD34, CD117 and CD133 (Fig. 3a). Analysis of CD73 and CD90 by flow cytometry confirmed the homogenous expression of mesenchymal marker genes within the whole cell population (Fig. 3b).

These findings, together with the fact that USCs adhere to plastic surfaces are consistent with previous reports [6, 17] and corroborate that USCs closely resemble MSCs.

### USCs have the potential to differentiate into osteogenic, chondrogenic and adipogenic lineages

**Fig. 4:**
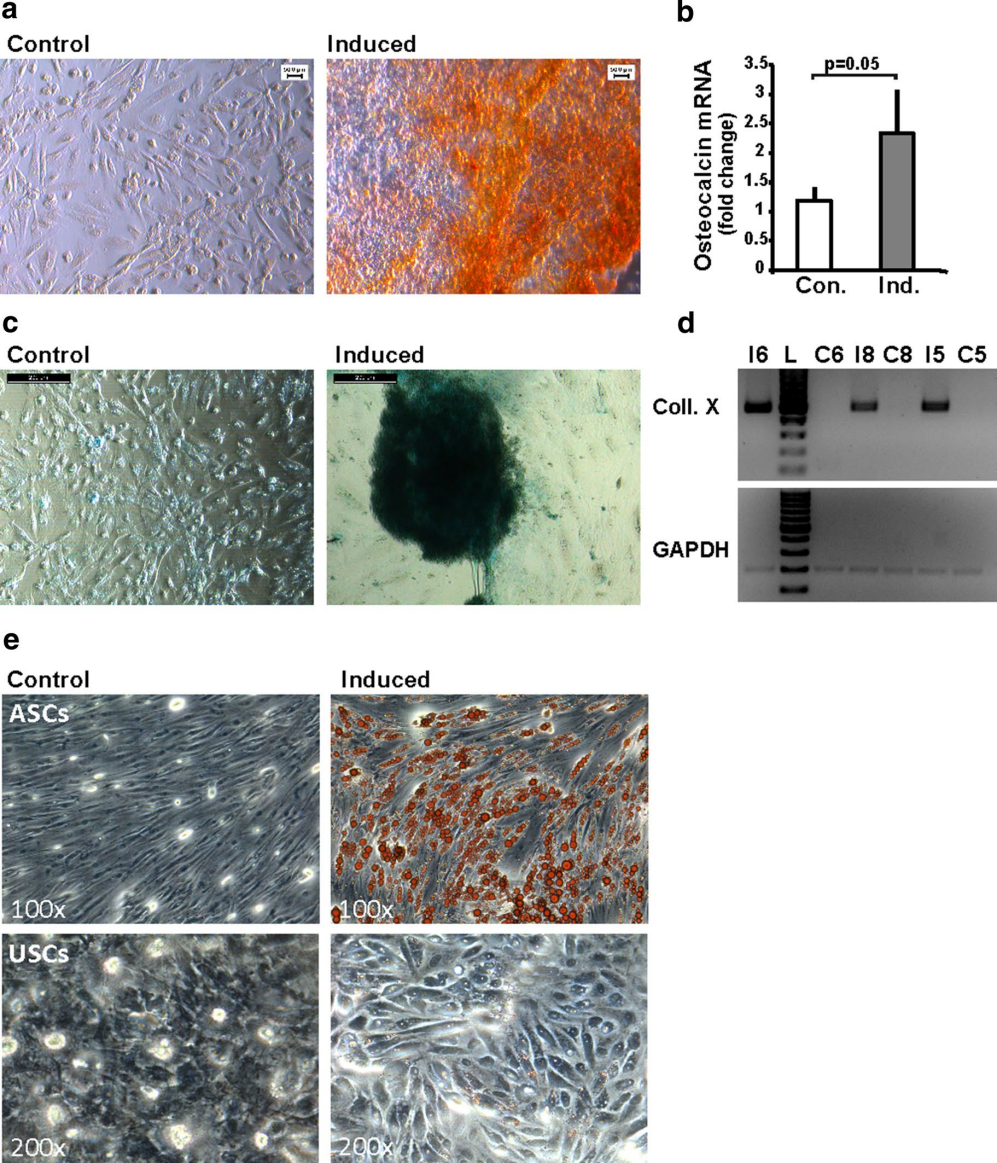
USCs have the potential to differentiate towards osteocytes, chondrocytes and adipocytes. **a** Osteogenic differentiation: After 28 days of culture in the induction medium, USCs stained positive for Alizarin red in contrast to the un-induced control. Representative images are shown. **b** mRNA expression of Osteocalcin relative to GAPDH was analyzed by qPCR. Induced cells showed a significant increase compared to the un-induced control (*p* = 0.05). *Error bars* indicate the standard deviation from three biological replicates (three different donors). *Con.* control, *Ind.* induced. **c** Chondrogenic differentiation: representative pictures showing morphological changes and alcian blue positive staining of USCs after 28 days of induction, compared to an un-induced control, are depicted. **d** The chondrogenic marker Collagen X (Coll. X) could only be detected in induced cultures by semi-quantitive PCR. GAPDH was used as loading control. Numbers represent different donors, *I* induced, *C* control, *L* 100 bp ladder. All bands are at the expected heights. **e** Adipogenic differentiation: USCs and ASCs were subjected to adipogenic differentiation for 24 days. Uninduced cells served as control. The formation of lipid droplets was visualized by Oil* Red* O staining. Representative images are shown

In order to further confirm that our USCs are functionally similar to MSCs and previously published USCs, we studied their potential to differentiate into osteoblasts, chondrocytes and adipocytes. Therefore, USCs were exposed to osteogenic induction medium for 4 weeks. At the end of this time period the cells aggregated and formed calcium precipitates, which were confirmed by alizarin red staining (Fig. 4a). On transcriptional level, osteocalcin mRNA expression showed a significant twofold increase in induced cells when they were compared to an uninduced control (*p* = 0.05) (Fig. 4b).

When USCs were cultivated with chondrogenic induction medium over a period of 28 days, cells formed aggregates as expected during differentiation, and stained positive for proteoglycans using Alcian blue (Fig. 4c). At mRNA level, differentiated USCs exhibited detectable levels of Collagen X in contrast to the non-differentiated control cells (Fig. 4d).

For adipogenic differentiation, USCs and ASCs as control were incubated for 24 days in adipogenic induction medium and stained with Oil Red O. Indeed, lipid droplets were detected in USCs compared to the uninduced control, although they were smaller and appeared much later during the course of differentiation than in ASCs (Fig. 4e). The reason might be that ASCs are derived from adipose tissue and are thereby already pre-conditioned to efficiently differentiate towards adipocytes.

These results demonstrate that USCs are indeed able to differentiate towards the osteogenic, chondrogenic and adipogenic lineages.

### USCs have immunomodulatory properties

**Fig. 5:**
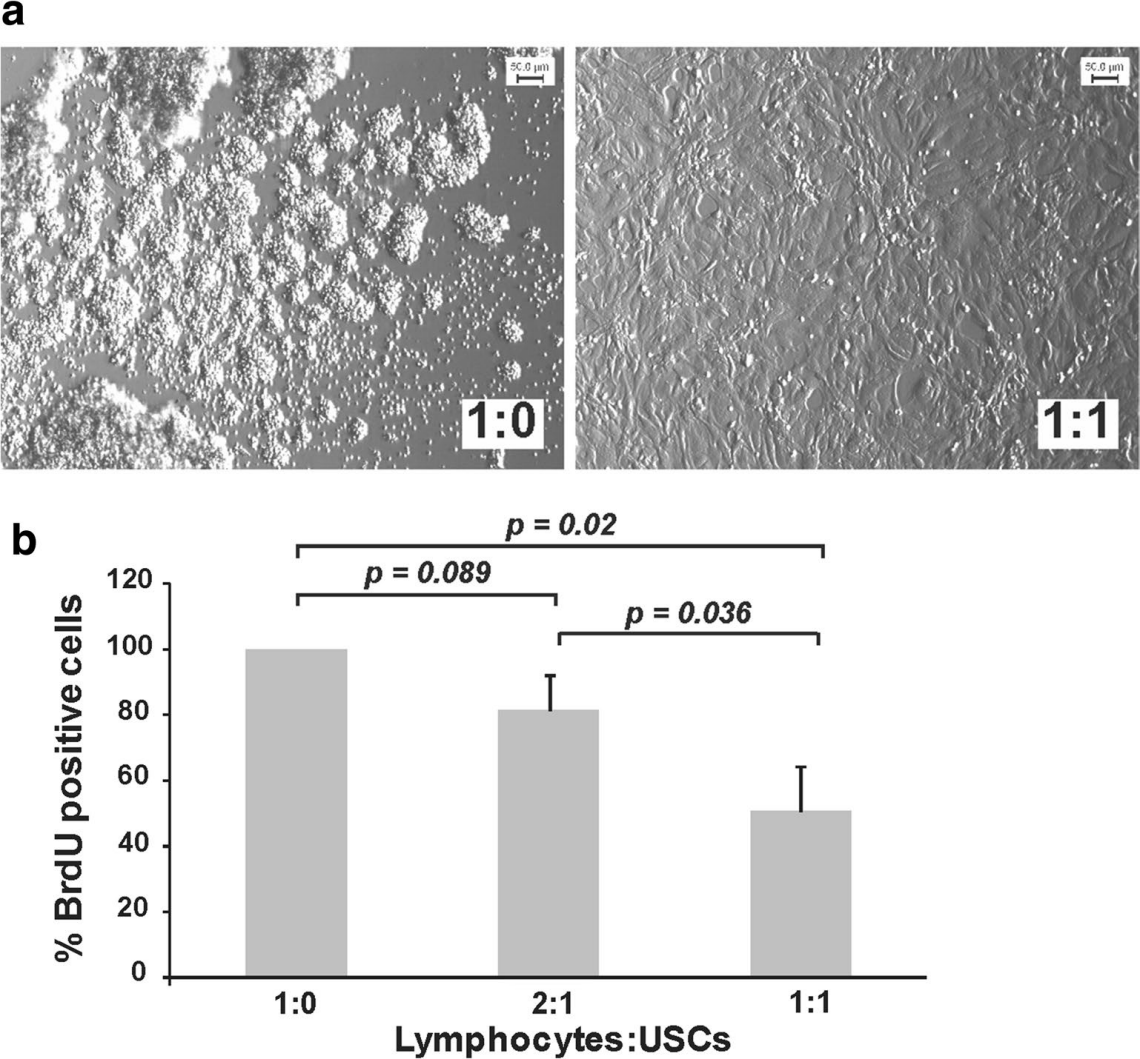
USCs display immunomodulatory properties. **a** The presence of USCs inhibited the typical cluster formation of lymphocytes after their activation by PHA. Representative pictures of lymphocytes alone (1:0) and lymphocytes and USCs at a 1:1 ratio are shown. **b** USCs reduced PHA-activated lymphocyte proliferation in a dose-dependent manner. *Error bars* indicate the standard deviation of biological triplicates

Finally, we wanted to test if USCs also display the typical immunosuppressive activity towards lymphocytes, which was to our knowledge not done before. Therefore, USCs were co-cultured with lymphocytes and after PHA stimulation the proliferation levels of lymphocytes were analyzed by measuring BrdU incorporation. Indeed, USCs inhibited the formation of cell clusters typically found after in vitro activation of lymphocytes [18] (Fig. 5a). Similarly, lymphocyte proliferation was significantly reduced by 50 % (*p* = 0.02) by co-culturing with USCs at a lymphocytes:USCs ratio of 1:1 (Fig. 5b).

These observations strongly suggest that USCs display typical characteristics of MSCs, including surface marker profile, differentiation potential as well as immunomodulatory properties.

### USCs from patients with EB

**Fig. 6:**
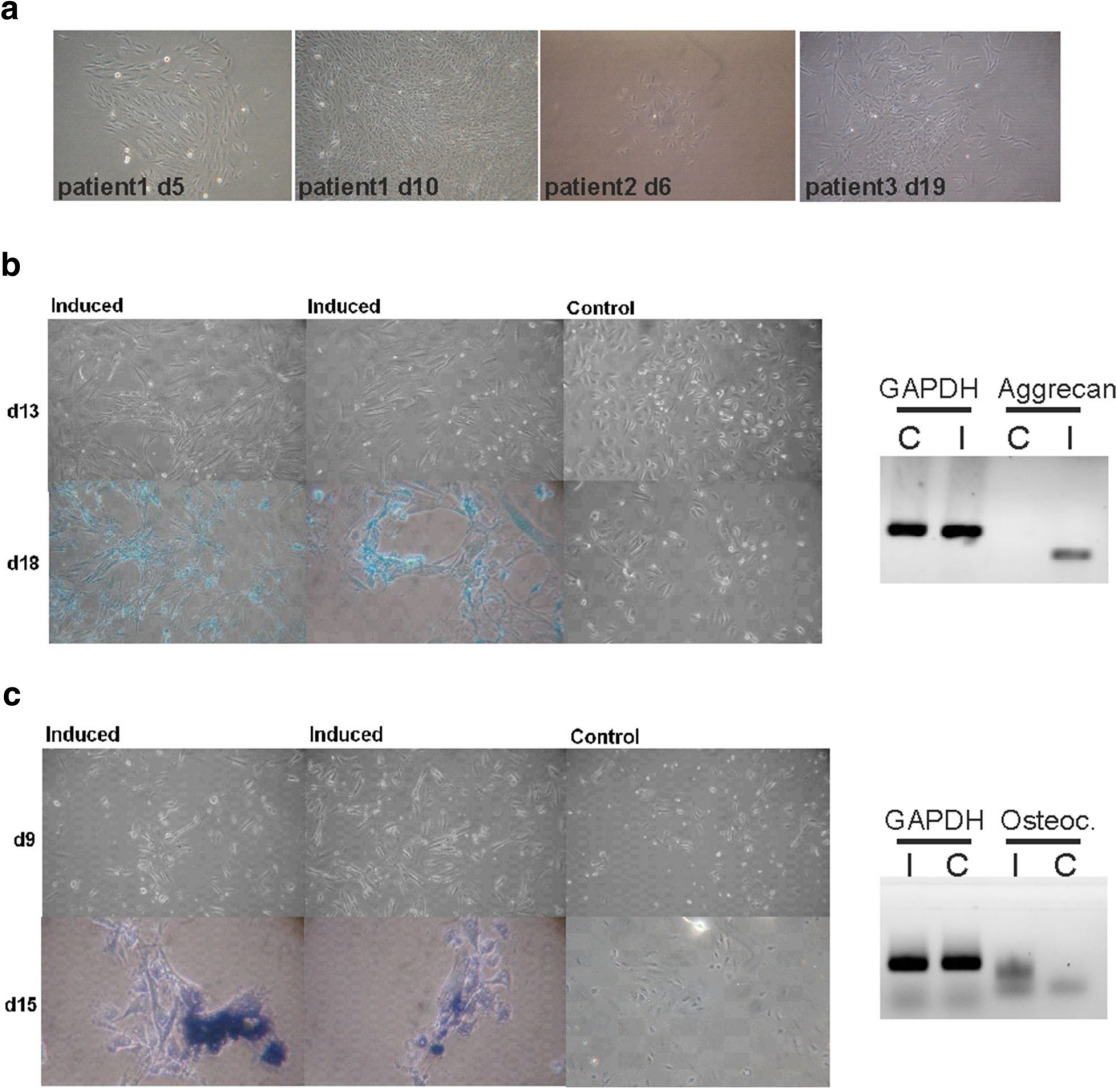
Isolation and differentiation of USCs from patients with EB. **a** Representative colonies of USCs isolated from patients with EB are shown. **b** Chondrogenic differentiation: representative pictures showing morphological changes and alcian* blue* positive staining of USCs after 18 days of induction, compared to an un-induced control, are depicted (*left*). The chondrogenic marker Aggrecan could only be detected in induced cultures by semi-quantitive PCR. GAPDH was used as loading control. *I* induced, *C* control (*right*). **c** Osteogenic differentiation: after 15 days of culture in the induction medium, EB-USCs stained positive for NBT/BCIP in contrast to the un-induced control. Representative images are shown (*left*). mRNA expression of Osteocalcin (Osteoc.) was analyzed by semi-quantitive PCR. Induced cells showed a significant increase compared to the un-induced control. GAPDH was used as loading control. *C* control, *I* induced (*right*)

After the optimization of USCs isolation and cultivation, as well as the characterization of USCs from healthy donors, we tested if our protocol was also applicable to samples from patients with epidermolysis bullosa in another laboratory (Fig. 6a). In total, 33 urine samples were obtained from 25 donors, suffering from different types of EB (dystrophic EB, junctional EB, EB simplex) (Additional file 1: Table S3). In contrast to urine samples from healthy volunteers, the average volume from patients with EB was only 58.3 ml, probably due to the fact that in some patients the urinary tract is also affected from blistering and scarring, rendering urination painful. Interestingly, the mean number of colonies was with 1.6 colonies per patient only slightly lower than the mean number of healthy volunteers (1.9). The number of samples showing cell proliferation was similar between both groups (60 % patients with EB, 58 % healthy volunteers) (Additional file 1: Table S3).

Also EB-patient derived USCs differentiated into chondrogenic and osteogenic lineages, as could be shown by positive alzian blue (chondrogenic lineages) or NBT/BCIP (osteogenic lineages) staining and morphologic characteristics of the respective lineages. Semi-qRT-PCR revealed the expression of lineage-specific genes compared to undifferentiated controls (Fig. 6b, c).

These data confirm that our optimized protocol is also applicable to the reproducible isolation of functional USCs from urine of patients with EB.

## Discussion

We describe here the isolation and characterization of cells from human urine termed USCs, which display similar markers, differentiation potential, adherence to plastic surfaces and immunomodulatory properties as adipose tissue derived mesenchymal stem cells (ASCs).

In contrast to the isolation of bulk urine cells and their reprogramming to induced pluripotent stem cells (iPSCs) recently described by us [19, 20], the direct isolation of USCs and their subsequent (trans-)differentiation in various cell lineages offers the advantage that transfection steps or the transduction with putatively oncogenic viruses can be completely omitted. However, since USCs have only a narrow differentiation potential compared to iPSCs, their usage for the generation of certain tissues, especially those originating from the ecto- and endoderm, is limited.

We and others already showed that USCs can be differentiated into the main mesenchymal lineages, such as chondrocytes, adipocytes and osteoblasts. Already fibroblasts could be beneficial for the treatment of patients suffering from dystrophic EB as a stable cell source for gene correction using COL7A1-carrying vectors. Furthermore, two recent reports indicate that application of MSCs improves wound healing [21] and transplantation efficiency [22] in patients with EB. However, recent reports demonstrate that ASCs, MSCs derived from adipose tissue, can also be trans-differentiated into keratinocyte-like cells just by co-incubation with conditioned medium or cultured keratinocytes. Furthermore, those cells were able to form a stratified structure similar to human skin on top of a decellularized dermal matrix [12]. Paunescu et al. demonstrated the differentiation of bone-marrow derived mesenchymal stem cells into keratinocytes by incubation with a mixture of different growth factors [13].

We therefore hypothesize that also USCs have the potential to trans-differentiate into keratinocyte-like cells, which could later be used for the engineering of transplantable skin-grafts by gene-therapy as therapeutic approach for patients with EB. The isolation of functional USCs from patients with EB, as shown here, is the first important step in this process.

Next, a reproducible protocol for the generation of therapeutically relevant amounts of keratinocytes from USCs is required, which might be hindered by their limited number, proliferation and differentiation potential. However, we are convinced to circumvent this problem by generating purer USCs-populations by flow cytometry or other enrichment strategies in the future. Furthermore, since USCs can be easily, inexpensively and painlessly isolated, the limited number of cells can be to some degree compensated by processing multiple samples.

## Conclusions

Summarized, the work presented here suggests a promising novel non-invasive route for obtaining stem cells from patients with EB, which might be used for therapeutic approaches after resolving the last open technical issues. Importantly, the retrieval of USCs might also be beneficial for the treatment of autoimmune disorders [23] and other diseases, where taking biopsies is painful.

## Additional file

10.1186/s13104-015-1686-7 Supplementary Tables 1–3.

## Abbreviations

ASCs: adipose tissue derived mesenchymal stem cells
EB: epidermolysis bullosa
iPSCs: induced pluripotent stem cells
MSCs: mesenchymal stem cells
USCs: urine-derived stem cells
