# Supplementary material for: Urine is a novel source of autologous mesenchymal stem cells for patients with epidermolysis bullosa
Source: BMC Res Notes. 2015 Dec 10;8:767. doi: 10.1186/s13104-015-1686-7 (PMC4676112; doi:10.1186/s13104-015-1686-7)
Supplement: Supplementary file 1 — 10.1186/s13104-015-1686-7 Supplementary Tables 1–3. [file 13104_2015_1686_MOESM1_ESM.pdf]

Supplementary tables:

Table S1. **Donor characteristics and isolation success of USCs from healthy donors.**

	Donors	Samples	Samples with cell proliferation (%)	Colonies formed* (± SD)	Urine volume in ml (± SD)
**Total**	21	127	59	2.1 ±1.4	202 ±77
**Male**	14	75	70	2.3 ±1.6	200 ±82
**Female**	7	52	42	1.6 ±0.8	203 ±72

*only successful isolations were considered

Table S2. **Comparison of the effect of different medium formulations on USCs.** The values represent the mean and standard deviation of at least biological triplicates, unless indicated otherwise.

	Colonies	Days until first colony appearance	First passage at day	Maximal population doublings
**pUSCs**	1.3 ±0.6	8.0 ±3.6	14.7 ±1.2	9.6 ±2.6
**Mesencult**	2.0 ±0.8	6.3 ±2.9	13.5 ±0.6	6.8 ±5.3
**Mesenpro**	1.7 ±1.2	6.3 ±2.9	14.0 ±3.5	1.7 ±1.2
**EGM-2**	2.0*	5.0*	11.0*	6.0*

*only one sample showed cell proliferation in EGM-2.

<u>Table S3.</u> Donor characteristics and isolation success of USCs from patients with EB. DEB: dystrophic EB, JEB: junctional EB, EBS: EB simplex.

	**Donors**	**Samples**	**Samples with cell proliferation (%)**	**Colonies formed***	**Urine volume in ml (± SD)**
**Total**	25	33	61	1.6	58
**DEB**	14	19	47	1.3	58
**JEB**	2	2	50	1.5	67.5
**EBS**	5	7	71	1.3	46
**Unknown**	4	5	100	2.2	61

*only successful isolations were considered
